# Defective sister chromatid cohesion is synthetically lethal with impaired APC/C function

**DOI:** 10.1038/ncomms9399

**Published:** 2015-10-01

**Authors:** Job de Lange, Atiq Faramarz, Anneke B. Oostra, Renee X. de Menezes, Ida H. van der Meulen, Martin A. Rooimans, Davy A. Rockx, Ruud H. Brakenhoff, Victor W. van Beusechem, Randall W. King, Johan P. de Winter, Rob M. F. Wolthuis

**Affiliations:** 1 Department of Clinical Genetics, section Oncogenetics, VU University Medical Center, Van der Boechorststraat 7, 1081 BT Amsterdam, The Netherlands; 2 Department of Epidemiology and Biostatistics, VU University Medical Center, De Boelelaan 1118, 1081 HV Amsterdam, The Netherlands; 3 Department of Medical Oncology, RNA Interference Functional Oncogenomics Laboratory, VU University Medical Center, De Boelelaan 1118, 1081 HV Amsterdam, The Netherlands; 4 Department of Otolaryngology—Head and Neck Surgery, VU University Medical Center, De Boelelaan 1118, 1081 HV Amsterdam, The Netherlands; 5 Department of Cell Biology, Harvard Medical School, 240 Longwood Avenue, Boston, Massachusetts 02115, USA

## Abstract

Warsaw breakage syndrome (WABS) is caused by defective DDX11, a DNA helicase that is essential for chromatid cohesion. Here, a paired genome-wide siRNA screen in patient-derived cell lines reveals that WABS cells do not tolerate partial depletion of individual APC/C subunits or the spindle checkpoint inhibitor p31^comet^. A combination of reduced cohesion and impaired APC/C function also leads to fatal mitotic arrest in diploid RPE1 cells. Moreover, WABS cell lines, and several cancer cell lines with cohesion defects, display a highly increased response to a new cell-permeable APC/C inhibitor, apcin, but not to the spindle poison paclitaxel. Synthetic lethality of APC/C inhibition and cohesion defects strictly depends on a functional mitotic spindle checkpoint as well as on intact microtubule pulling forces. This indicates that the underlying mechanism involves cohesion fatigue in response to mitotic delay, leading to spindle checkpoint re-activation and lethal mitotic arrest. Our results point to APC/C inhibitors as promising therapeutic agents targeting cohesion-defective cancers.

## Abstract (web summary)

Cohesion is associated with many forms of cancer. De Lange *et al*. show that such cohesion defects can sensitise cells to apoptosis in response to a new APC/C ubiquitin ligase inhibitor, by prolonging mitotic arrest and checkpoint activation due to cohesion fatigue.

## Graphical abstract

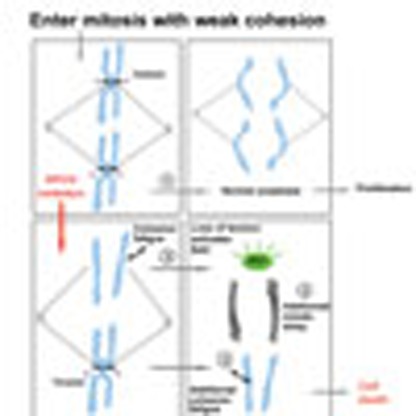

Cell division requires the duplication of all chromosomes, followed by their segregation as two identical sister chromatids into two new daughter cells. Sister chromatid cohesion holds sister chromatids together until their proper separation is initiated at the metaphase-to-anaphase transition. Pairing of sister chromatids is achieved by a huge ring-shaped protein complex named cohesin, which consists of Smc1, Smc3, Rad21 (Scc1 in yeast) and either SA1 or SA2 (Scc3 in yeast). Besides keeping sister chromatids paired during early stages of mitosis, cohesin's DNA tethering capacity facilitates multiple additional processes in the cell, such as DNA repair, ribosome biogenesis, regulation of gene transcription and initiation of DNA replication^1^. Defects in the cohesion network are the cause of several rare genetic diseases named cohesinopathies. These include Cornelia de Lange Syndrome (CdLS, caused by mutations in NIPBL, Smc1A, Smc3, Rad21 or HDAC8 (refs 2, 3, 4, 5)), Roberts Syndrome (RBS, caused by ESCO2 mutations^6^,^7^) and Warsaw Breakage Syndrome (WABS, caused by DDX11 mutations^8^). Although it is not clear whether these predispositions are linked to an increased cancer risk, mutations in genes encoding cohesin subunits and regulators have been reported in a substantial number of human tumours^9^,^10^,^11^,^12^,^13^,^14^,^15^. Cohesion defects may thus form a new hall mark of cancer that could be exploited in therapy.

When cells enter mitosis, the bulk of cohesin is removed from chromosome arms during prophase, in a manner dependent on phosphorylation of cohesin subunits by mitotic kinases and the cohesion antagonist Wapl (reviewed in ref. 16). However, centromeres are protected against loss of cohesion by Sgo1, which attracts a phosphatase to prevent phosphorylation of the Wapl antagonist Sororin, and SA2 (refs 17, 18, 19, 20, 21). During prometaphase, the kinetochores of paired sister chromatids attach to the mitotic spindle and subsequently come under tension of spindle pulling forces. Resisting spindle pulling forces is an important function of sister chromatid cohesion, preventing premature sister chromatid separation until the last pair of sister chromatids becomes bioriented on the mitotic spindle. The occurrence of prematurely separated sister chromatids which lose microtubule-kinetochore attachments activates the spindle assembly checkpoint (SAC)^22^. Continuous arrest of cells in the SAC may lead to cell death or highly aneuploid daughter cells^23^.

The SAC is an evolutionary conserved signalling cascade that acts in prometaphase and keeps cyclin B1-Cdk1 active during the process of chromosome biorientation^24^,^25^. Proper attachment of all the paired sister chromatids to the spindle and their alignment to the cell equator is a stochastic process that can take roughly up to 1 h in normal cells. Maintenance of cyclin B1-Cdk1 activity during this phase is essential to keep the mitotic state until biorientation is complete. Simultaneously, Separase, a Rad21 protease, must be kept inactivated to protect centromere cohesion. The SAC is kept activate by kinetochores that are not properly attached to spindle microtubules, stimulating production of the mitotic checkpoint complex (MCC), composed of BubR1, Bub3, Mad2 and Cdc20 (ref. 26). The MCC blocks the anaphase promoting complex or cyclosome (APC/C), a multi-subunit E3 ubiquitin ligase, so that three of its substrates remain stable for multiple hours: Securin, which blocks Separase^27^, cyclin B1, which keeps Cdk1 active to keep cells in mitosis^28^, and geminin, which blocks premature DNA replication licensing^29^. Achievement of proper attachment and centromere tension silences the SAC, activating APC/C-Cdc20. This leads to degradation of securin to release Separase, cleaving the cohesin subunit Rad21 and allowing chromatid separation to opposite spindle poles. Cyclin B1 degradation occurs at the same time and causes inactivation of Cdk1, initiation of cytokinesis and mitotic exit^30^. Geminin is also degraded, preparing cells for DNA replication^29^.

SAC silencing may involve multiple mechanisms, such as tension-sensitive kinetochore phosphorylations^31^, activation of phosphatases that antagonize certain mitotic kinases^32^ and dynein-microtubule-mediated stripping of SAC proteins from kinetochores upon microtubule attachment^33^. Furthermore, p31^comet^ promotes the release of Mad2 from the MCC, thereby initiating Cdc20 release downstream of kinetochores^34^,^35^,^36^,^37^.

Cancer arises by an accumulation of genetic and epigenetic alteration in cancer genes, disturbing the normal signalling routes in the cell. This can make tumour cells highly dependent on a specific pathway that remains intact, while in healthy cells the backup pathway still exists. The phenomenon that two genes or two signalling pathways can compensate each other, but inactivation of both diminishes cell viability, is called ‘synthetic lethality'. Such interactions between pathways can be exploited to eradicate tumour cells without many side effects on normal tissues^38^. Here, we aim to identify pathways that are specifically lethal in combination with defects in sister chromatid cohesion, to start to develop of a new targeted cancer therapy. We use a patient fibroblast cell line in which DDX11 mutations cause cohesion defects^8^ in parallel with its functionally corrected counterpart as model system and subject these cell lines to siRNA screens in order to find lethal interactors. We find that the DDX11 mutant cells are hypersensitive to inhibition of the APC/C. APC/C inhibition to a level that is tolerated by normal cells, causes a detrimental further loss of chromatid cohesion during mitosis in cohesion-defective cells, and subsequently induces mitotic death. This lethality is observed in a range of different cohesion-defective cells and requires a functional SAC. In line with this observation, treatment with the recently published cell-permeable APC/C-inhibiting drug apcin is particularly toxic in cell lines with defective sister chromatid cohesion, including tumour cell lines.

## Results

### A genome-wide siRNA screen in DDX11 mutant cells

**Figure 1:**
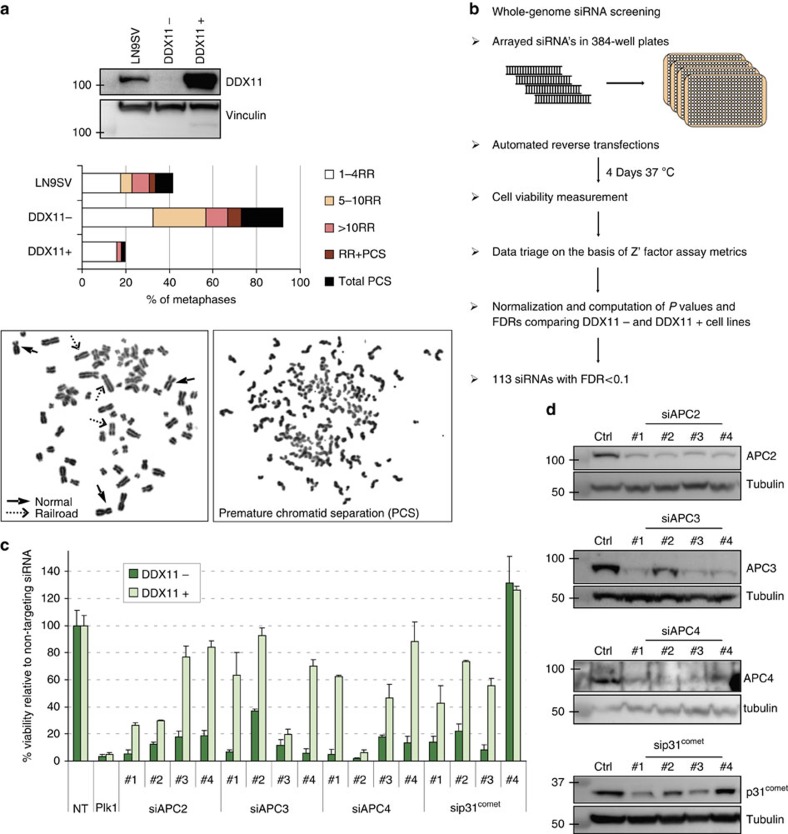
Genome-wide siRNA screen in DDX11 mutant cells. (**a**) Western blot and cohesion defect analysis of DDX11^−^ and DDX11^+^ cells next to SV40-immortalized wild-type LN9SV fibroblasts. Combined data of two independent metaphase preparations are shown. Two examples of two metaphases illustrate normal and railroad (RR) chromosomes as well as PCS. (**b**) Schematic representation of the genome-wide siRNA screening procedure using Dharmacon's siARRAY whole human genome siRNA library. Three independent siRNA screens for both cell lines were performed. (**c**) The same platform was used to perform two independent deconvolution experiments with a selection of 98 hits in both cell lines, of which the results of four genes are shown. Error bars denote standard deviations of two independent experiments. (**d**) Cells were transfected with the indicated siRNAs and protein levels were analysed after 3 days by western blot. PCS, premature chromatid separation.

We generated SV40-immortalized fibroblasts derived from a WABS patient and functionally corrected the cohesion defects in this cell line (railroad chromosomes, RR, and premature sister chromatid separations, PCS, Fig. 1a, ref. 8) by stable transfection of DDX11 cDNA (Fig. 1a, ref. 8). We used these two cell lines, hereafter named DDX11^−^ and DDX11^+^ cells, to screen for genes whose inactivation is specifically lethal in cohesion-defective cells by performing whole-genome siRNA screens. An overview of the procedure is provided in Fig. 1b. Briefly, cells were reverse transfected in 384-well plates with single-target pools of four distinct siRNAs using an automated platform and viability was measured after 4 days using the CellTiter-Blue assay. We computed *P* values and false discovery rates (FDR) for the difference in cell viability with each siRNA between the two cell lines (Supplementary Fig. 1). This revealed 113 siRNAs with FDR<0.1. We excluded 32 genes based on updated library annotation according to NCBI RefSeq58 or because they exhibited the highest lethality in DDX11^+^ cells and cherry-picked 17 additional genes with FDR slightly above the threshold, so in total 98 hits were selected (Supplementary Data 1).

We rescreened these 98 hits in DDX11^−^ and DDX11^+^ cells with deconvoluted sets of 4 siRNAs. Subsequently, we calculated the differential effect (ratio DDX11^+^/DDX11^−^) and toxicity (lethality in DDX11^+^ cells) for every individual siRNA (Supplementary Data 2). Interestingly, of 19 genes showing ratio >2 and toxicity <50%, we identified *APC2*, *APC3/Cdc27* and *APC4*, which encode three different components of the APC/C. Moreover, we also identified *Mad2L1BP/p31*^comet^, which encodes a negative regulator of the APC/C inhibitor Mad2. Accompanying western blots (Fig. 1c,d) suggested that the weaker effects of siAPC3#2 and sip31^comet^#4 in DDX11^−^ cells result from lower knockdown efficiency. The toxicity of siAPC2#1, siAPC2#2, siAPC3#3 and siAPC4#2 in DDX11^+^ cells might relate to an induction of off-target effects by these RNAi oligos. The increased sensitivity of DDX11^−^ cells to APC2 inhibition was confirmed with an additional pool of four unrelated APC2 targeting siRNAs (Supplementary Fig. 2). Together, these results indicate that DDX11 mutant cells are highly sensitive to knockdown of APC/C subunits.

### Cohesion defects sensitize to APC/C inhibition

**Figure 2:**
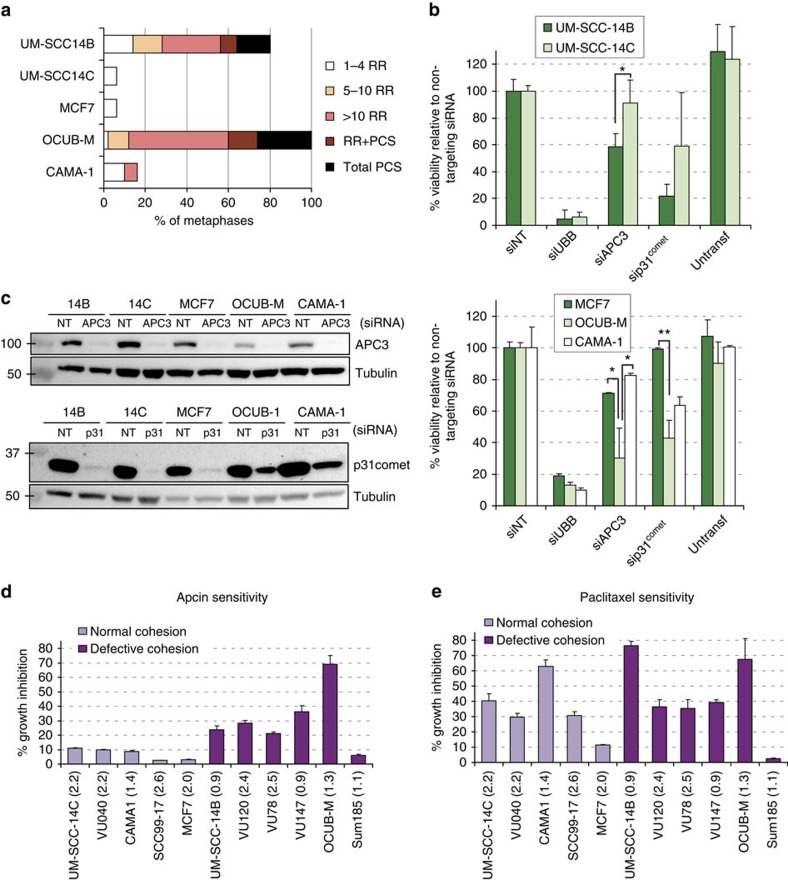
Defective sister chromatid cohesion sensitizes to APC/C inhibition. (**a**) Cohesion defect analysis of five tumour cell lines (**b**,**c**) Cells were transfected with the indicated siRNAs. Protein levels were analysed after three days by western blot and cell viability was measured after 5 days using a CellTiter-Blue assay. Error bars denote s.d. of at least three technical replicates. (**d**,**e**) A panel of tumour cell lines with known cohesion status was seeded at optimized densities in two 96-wells plates. The next day, viability was assayed in one plate (t=0), whereas in the other plate medium was replaced with medium containing DMSO, apcin (150 μM) or paclitaxel (2 nM) and viability was measured 3 days later. We calculated the percentage growth inhibition of treated versus untreated cells and corrected this for the number of cell divisions (indicated between brackets) in untreated cells during the experiment. Error bars denote s.d. of at least three technical replicates. Apcin sensitivity was significantly higher (*P*=0.030) in cohesion-defective cells as compared with cells without cohesion defects, whereas paclitaxel sensitivity did not differ (*P*=0.537) using a Wilcoxon rank-sum test.

We investigated the response to APC/C inhibition in a number of cell lines from our laboratory with known cohesion status. Two head and neck squamous cell carcinoma (HNSCC) cell lines, isolated from a single patient, represent a highly related panel of cells. Importantly however, they differ in their sister chromatid cohesion status. Sister chromatid cohesion is normal in UM-SCC-14C but disturbed in UM-SCC-14B^39^ (Fig. 2a). Of three luminal-type breast cancer cell lines, OCUB-M cells exhibit severe cohesion defects, whereas most metaphases of MCF7 and CAMA-1 cells appear normal (Fig. 2a). Depletion of APC3 or p31^comet^ induced the strongest growth inhibition in UM-SCC-14B and OCUB-M cells (Fig. 2b,c). Similarly, APC/C knockdown showed a stronger effect in ESCO2^−^ cells, derived from a RBS patient^40^, as compared with its functionally corrected counterpart ESCO2^+^ (Supplementary Fig. 3). We conclude that increased sensitivity to APC/C inhibition is not restricted to DDX11 mutant cells, but could be a more general feature of cells in which the cohesion of sister chromatids during metaphase is weak.

Interestingly, a new APC/C-inhibiting compound named apcin has recently been developed^41^, which partially inhibits APC/C activity. Apcin acts by competitively binding to the mitosis-specific APC/C cofactor Cdc20 and hampering the ubiquitination of D-Box containing substrates^41^. As expected, apcin phenocopied the effects of the APC/C^Cdc20^ impairing siRNAs in the DDX11^−^ and DDX11^+^ cell panel (Supplementary Fig. 4). We then analysed a larger panel of HNSCC and luminal breast cancer cell lines of which metaphase spreads had been analysed in our laboratory. Sensitivity to apcin was corrected for the number of cell divisions during three days treatment. This revealed a remarkable and significant correlation between the presence of cohesion defects in tumour cells and sensitivity to apcin (Fig. 2d). Interestingly, the cohesion status did not correlate well with sensitivity to paclitaxel (Fig. 2e), which activates the SAC by interfering with microtubule dynamics and spindle forces.

### APC/C inhibition aggravates cohesion defects and causes mitotic death

**Figure 3:**
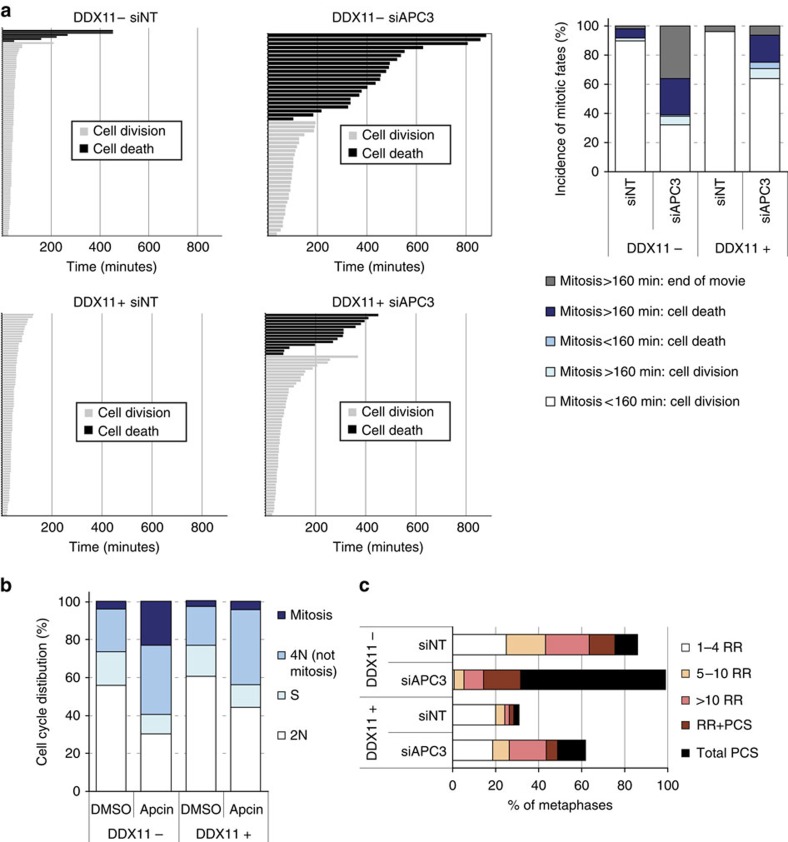
APC/C inhibition leads to a lethal mitotic delay and further loss of cohesion. (**a**) DDX11^−^ and DDX11^+^ cells were transfected with the indicated siRNAs. Two days after transfection, cells were analysed for 16 h using live cell imaging to measure the duration of mitosis from the time of NEB until anaphase or (if no anaphase was visible) viable mitotic exit, or cell death. Mitosis duration significantly correlated with mitotic fate; *P*=2.21*10^−8^ in DDX11^−^ cells and *P*=4.67*10^−5^ in DDX11^+^ cells, using a students *t*-test. (**b**) DDX11^−^ and DDX11^+^ cells were treated with 150 μM apcin for 24 h and analysed by flow cytometry. (**c**) DDX11^−^ and DDX11^+^ cells were transfected with APC3 siRNA for 3 days and metaphases were analysed for cohesion defects. Means of two independent experiments are shown. NEB, nuclear envelope breakdown.

Apparently, cohesion defects make cells particularly vulnerable to a delay in mitosis when APC/C activity is reduced. We used time-lapse microscopy in order to analyse the mitotic events related to this sensitivity in live cells as they progressed through mitosis. Microscopic fields were analysed for 16 h and mitosis durations from nuclear envelope breakdown (NEB) to anaphase or cell death are shown in Fig. 3a. This shows that the duration of mitosis strongly correlates with cell death. Moreover, a larger percentage of APC3 depleted DDX11^−^ cells undergo mitotic death as compared with APC3 depleted DDX11^+^ cells. This difference is probably larger than displayed, as many DDX11^−^ cells were still in mitosis at the end of the movie (Fig. 3a bar graph). In line with these observations, consecutive flow cytometry analyses (Supplementary Fig. 5a–c) revealed an increased mitotic fraction in DDX11^−^ cells (day 2) that is followed by a strong induction of a fraction with 4N DNA content that stain negative for phospho-Histone H3 (day 3), probably representing mitoses that do not produce two new cells because of cytokinesis failure. APC/C inhibition using different siRNAs shows comparable results (Supplementary Fig. 5d). Importantly, 24 h apcin treatment also specifically blocked DDX11^−^ cells in mitosis (Fig. 3b). We then performed a cohesion defect analysis, which revealed a striking enhancement of premature chromatid separation upon APC3 knockdown in DDX11^−^ cells (Fig. 3c).

**Figure 4:**
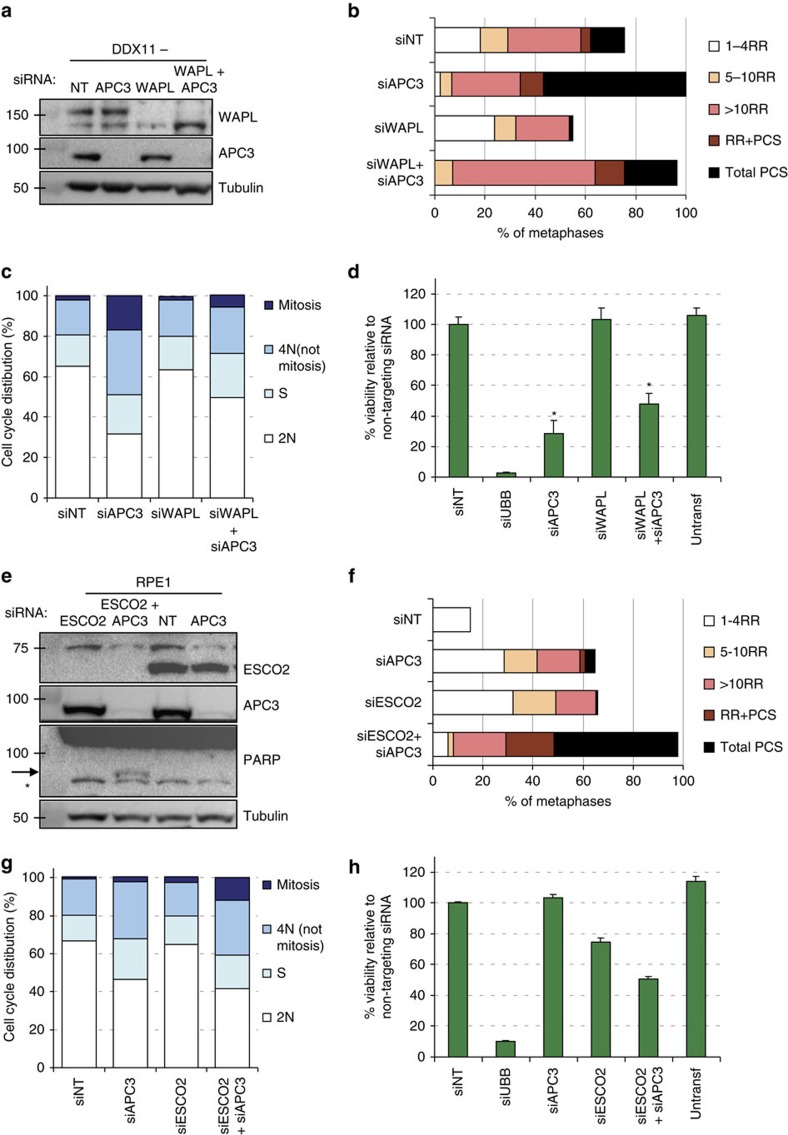
Cohesin levels determine sensitivity to APC/C inhibition. (**a**–**d**) DDX11^−^ cells were first transfected with either non-targeting or WAPL siRNA, to obtain an efficient knockdown. Two days later, cells were again transfected with the indicated siRNAs. At day 3 after the second transfection round, cells were harvested for western blot (**a**), cohesion defect analysis (**b**), and flow cytometry (**c**). At day 4, viability was measured with a CellTiter-Blue assay (**d**). Viability upon siAPC3 only was significantly different from siWAPL+siAPC3 (*P*=0.002 using a students *t*-test), indicated by an asterisk. (**e**–**h**) RPE1 cells were transfected with the indicated siRNAs. Western blot (**e**) cohesion defect analysis (**f**) and flow cytometry (**g**) were performed 2 days after transfection. Means of two independent experiments are shown. The asterisk indicates non-specific background staining. At day 4, viability was measured with a CellTiter-Blue assay (**h**). A students *t*-test revealed *P*=0.057 when comparing viability upon siAPC3 only and siESCO2+siAPC3. Error bars denote standard deviations of three technical replicates.

We reasoned that such severe cohesion defects might explain the observed cell death. If that is indeed the case, directly reinforcing cohesion during metaphase should rescue cohesion, mitotic progression and viability. To test this, we used an siRNA targeting WAPL, a protein required to remove the majority of cohesin complexes from chromosome arms during prophase^42^,^43^. Indeed, WAPL knockdown partially restored sister chromatid cohesion in DDX11^−^ cells and also reverted the accumulation of mitotic cells and lethality upon APC3 knockdown (Fig. 4a–d). We then asked whether the opposite was also true: can artificially weakening sister chromatid cohesion in otherwise normal cells sensitize them to lethal APC/C inhibition? Indeed, co-depletion of ESCO2 and APC3 in RPE1 cells resulted in severe cohesion defects, increased mitotic delay, caspase-dependent PARP cleavage (indicative of apoptosis induction) and reduced cell viability (Fig. 4e–h). Co-depletion of Rad21 and APC3, or of DDX11 and APC3, gave the same results (Supplementary Fig. 6). Notably, acute knockdown of DDX11 in RPE1 cells did not alter APC3 levels and by using varying concentrations of siAPC3 we also excluded that small differences in APC3 expression underlie the differential sensitivity of DDX11^−^ and DDX11^+^ cells (Supplementary Fig. 7).

In conclusion, our findings indicate that weakened sister chromatid cohesion at the start of mitosis, together with a reduction of APC/C activity, induce prolonged mitosis, massive premature chromatid separation and mitotic death.

### Weak cohesion plus APC/C inhibition leads to cohesion fatigue

**Figure 5:**
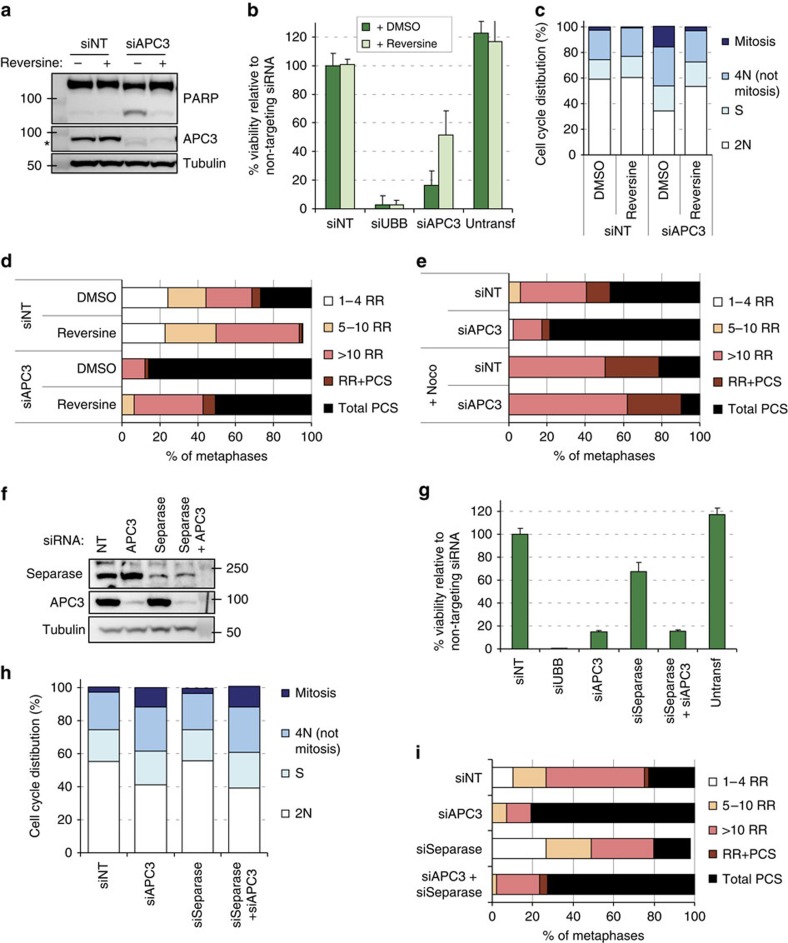
Cohesion fatigue explains the synergy of weak cohesion and APC/C inhibition. (**a**–**d**) DDX11^−^ cells were transfected with the indicated siRNAs and after 1 day 100 nM reversine or DMSO was added. Western blot, flow cytometry and cohesion defect analysis were performed two days after transfection and cell viability was measured four days after transfection. The asterisk indicates detection of residual cleaved PARP signal in the APC3 blot. (**e**) DDX11^−^ cells were depleted of APC3 for 30 h. After shaking off mitotic cells, cells were incubated with 100 ng ml^−1^ nocodazole for an additional 14 h and analysed by cohesion defect analysis. (**f**–**i**) DDX11^−^ cells were transfected with the indicated siRNAs. Western blot, flow cytometry and cohesion defect analysis were performed 2 days after transfection and cell viability was measured four days after transfection. Representatives of two independent experiments are shown. Error bars denote standard deviations of at least three technical replicates.

To investigate whether the observed cell death was strictly related to mitosis, we accelerated mitosis by blocking the spindle checkpoint using the Mps1 inhibitor reversine^44^ (Fig. 5a–d; the experiment including DDX11^+^ cells is shown in Supplementary Fig. 8). Indeed, reversine reduced caspase-dependent PARP cleavage and partially rescued cell viability in response to APC3 knockdown (Fig. 5a,b). Furthermore, the increase of mitotic fraction and cohesion defects (Fig. 5c,d) was strongly reduced. These results show that a spindle checkpoint-dependent arrest contributes to lethality by APC/C inhibition in cohesion-defective cells. This is in line with previous reports showing that the mitotic arrest induced by APC/C inhibition is dependent on activation of the spindle checkpoint^22^,^45^. This mechanism involves a phenomenon known as ‘cohesion fatigue'; a gradual loss of sister chromatid cohesion that can be observed during a prolonged mitosis^46^,^47^. Importantly, cohesion fatigue is thought to largely depend on microtubule pulling forces. We therefore used nocodazole to block the development of tension across sister kinetochores. Indeed, APC3 knockdown did not further increase the cohesion defects in DDX11^−^ cells in the absence of a functional mitotic spindle (Fig. 5e). We then investigated whether residual activity of Separase, the enzyme that becomes activated upon APC/C-mediated Securin degradation^48^, is also responsible for additional cohesion loss. In line with the reported APC/C and Separase independence of cohesion fatigue^46^, the effects of APC3 knockdown on cohesion, cell cycle and viability did not change when Separase was co-depleted (Fig. 5f–i).

**Figure 6:**
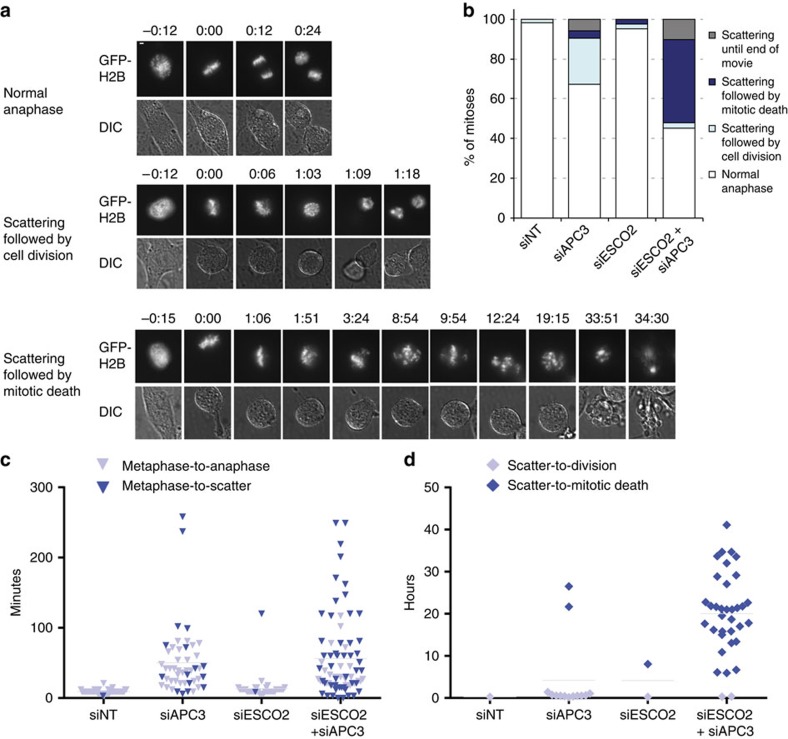
Severely prolonged mitosis with scattered chromosomes results in mitotic death. RPE1 cells were imaged every 3 min for 48 h using time-lapse microscopy, starting two days after transfection with the indicated siRNAs. (**a**) Representative examples of the observed phenotypes ‘normal anaphase' (siESCO2), ‘scattering followed by cell division' (siAPC3) or ‘scattering followed by mitotic death' (siESCO2+siAPC3). Bar 5 μM. (**b**) Percentages of mitotic fates observed in the different conditions (siNT *N*=55, siAPC3 *N*=52, siESCO2 *N*=42, siESCO2+siAPC3 *N*=64). (**c**) Duration (in minutes) from the time of metaphase plate formation until either a normal anaphase or chromosome scattering was observed. (**d**) Duration (in hours) until the cells with scattered chromosomes (the dark blue cells in (**c**)) show either cell division or mitotic death.

To visualize sister chromatid alignment at the metaphase plate, we then used GFP-H2B expressing RPE1 cells and analysed chromosome congression by time-lapse fluorescence microscopy (Fig. 6 and Supplementary Movies 1–6). Combined depletion of ESCO2 and APC3 caused a high percentage of mitoses to lose chromosome alignment on the metaphase plate, a process we termed chromosome scattering. The majority of these cells arrested in mitosis for many hours, and in almost all cases where APC3 RNAi was combined with ESCO2 RNAi, the prolonged mitotic arrest culminated in cell death. Single APC3 knockdown also increased metaphase duration, but this was in most cases followed by a normal anaphase and cell division, although in some cells the chromosomes also left the metaphase plate prior to the start of anaphase. In such cases, APC3 RNAi cells eventually divided perpendicular to the culture dish (Supplementary Movies 3,4), which indicated that the spindle had rotated, an effect sometimes observed after APC/C inhibition^49^. In addition, increased microtubule detachment from kinetochores may lead to a similar phenotype, for example resulting from impaired APC/C-dependent cyclin A degradation^50^. This does not necessarily induce a permanent mitotic arrest, but may still allow gradual cyclin B degradation and eventually mitotic exit, which would also explain cell division following scattering under conditions when only APC3 is depleted. It seems reasonable, however, that the more severe chromosome scattering observed in cohesion-defective cells upon depletion of APC3 largely reflects the premature chromatid separation that we observed in our cohesion defect analyses of fixed cells (Fig. 4f). In summary, when APC3 is depleted, cells delay in metaphase, which can lead to some scattering of the chromosomes away form the metaphase plate, but this eventually leads to anaphase and cytokinesis (Fig. 6c,d). However, when APC3 is depleted under conditions of impaired sister chromatid cohesion, a form of scattering is observed that relates to cohesion fatigue and causes cell death.

## Discussion

Mitotic cells require sister chromatid cohesion for maintaining a physical connection between replicated DNA molecules, to resist pulling forces and allow chromosome biorientation. Mutations in this network may facilitate tumorigenesis, possibly because they increase the chance of acquiring further genetic alterations^10^. However, such defects might also be disadvantageous in specific conditions, which would provide an opportunity to target those tumours. Regardless, cohesion defects are normally not observed in healthy cells, but can be detected in many tumours, thereby forming an interesting new target for cancer therapy. Here, we show that impaired sister chromatid cohesion, which is in itself not fatal, could become particularly detrimental when a cell encounters a substantial reduction of APC/C activity. It is important to note that a complete abolishment of APC/C activity is lethal in all cells (reviewed in ref. 51), but, perhaps depending on cellular context, cells may well tolerate reduced APC/C activity^52^,^53^,^54^. Our synthetic lethality screen revealed enhanced sensitivity of DDX11 mutant cells for reduced protein expression of different subunits of the APC/C, as well as the Cdc20 activating p31^comet^ that silences the mitotic checkpoint. We did not identify additional APC/C subunits in the RNAi screen, which may be due to incomplete knockdown and effects that fall outside the window of differential tolerance, or from the induction of lethal off-target effects by some siRNAs. Importantly, enhanced sensitivity to APC/C inhibition was further validated in several additional cell lines with weakened sister chromatid cohesion. This indicates that it should be possible to pinpoint a discriminative level of APC/C inhibition that is therapeutically relevant.

**Figure 7:**
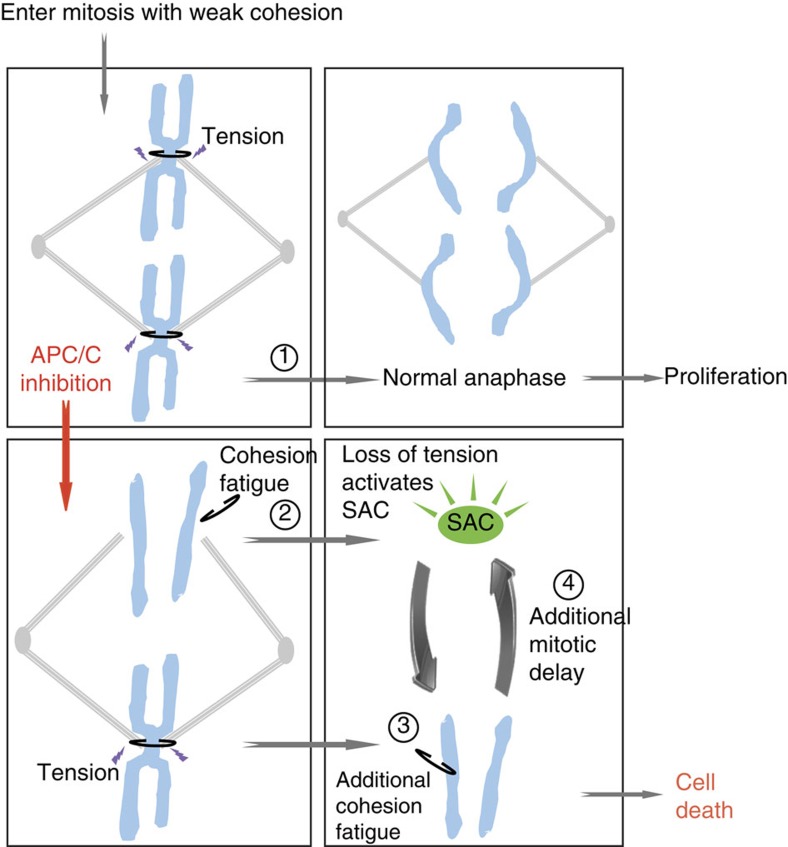
Model. Cells entering mitosis with weakened cohesion normally manage to resist spindle tension until anaphase (1). Partial APC/C inhibition slows down cyclin B1 degradation and causes a prolonged mitosis with an intact mitotic spindle. During this prolonged mitosis, a gradual loss of sister chromatid cohesion may occur, that is dependent on microtubule pulling forces. This may cause some chromosomes to lose functional cohesion, resulting in ‘cohesion fatigue' (2). Loss of tension on the kinetochores of those prematurely separated chromosomes then re-activates the SAC, leading to full inhibition of the APC/C and further blockage of mitotic exit. This can cause additional sister chromatid pairs to undergo cohesion fatigue (3), which in turn keeps the SAC activated (4). Eventually, this will result in death in mitosis or aberrant exit from mitosis as one or more aneuploid cells.

We propose a model in which the enhanced sensitivity of cohesion-defective cells to APC/C activity involves the previously reported appearance of unscheduled chromatid separation during a metaphase arrest, termed ‘cohesion fatigue'^46^ (Fig. 7). Although all cells face a prolonged mitosis when APC/C activity is partially reduced, most normal cells will eventually manage to sufficiently reduce cyclin B1 and Securin levels, allowing normal cell division scheduled in synchrony with anaphase. However, cells with impaired cohesion at the start of mitosis will sooner reach the point at which chromatid connections on one or more paired sister chromatids become insufficient to resist spindle pulling forces. The resulting premature sister chromatid separation and concomitant loss of tension and attachment to the spindle will re-activate the SAC, or prevent its timely inactivation^55^. This in turn feeds forward to block APC/C^Cdc20^ activity more effectively, which prolongs mitosis even more and increases the chance of additional paired sister chromatids losing cohesion. Eventually, many of these cells die in mitosis, or exit mitosis as non-productive daughter cells.

The cellular responses to mitotic delay are widely variable and appear to depend on an intriguing molecular competition between pathways leading to either apoptosis or slippage^23^,^56^. Our findings suggest that the outcome of this race is strongly influenced by the level of sister chromatid cohesion at the start of mitosis, and the residual activity of the APC/C in prometaphase. We propose that under conditions of further APC/C inhibition, slippage is prevented and cells eventually die by apoptosis due to a more severe mitotic blockade.

APC/C activity exerts both pro- and anti-proliferative effects, which complicates its use as a target in cancer therapy. The differential oscillation of its co-activators Cdc20 and Cdh1 confers an important level of APC/C regulation^57^. Although APC/C^Cdc20^ drives mitosis, APC/C^Cdh1^ is mainly involved in maintaining the G0/G1 state. It has been suggested that inactivation of APC/C^Cdh1^ might contribute to cancer growth, through stabilization of oncogenic substrates that fuel proliferation^53^. Indeed, Cdh1^−/+^ mice are more susceptible to spontaneous tumours^58^. Therefore, it seems desirable to exclusively inhibit APC/C^Cdc20^. This argues for a strategy of APC/C^Cdc20^ inhibition, such as exemplified by the novel APC/C inhibitor apcin, in favour of one that inhibits both APC/C^Cdch1^ and APC/C^Cdc20^, for example by proTAME, a drug which generally prevents cofactor binding to the APC/C. Interestingly, combining apcin with proTAME synergistically blocks mitotic exit^41^. This may relate to a similar mechanism as described above: in cells without pre-existing cohesion defects, APC/C inhibition resulting from combined treatment may be sufficiently strong to arrest cells in mitosis long enough to cause cohesion fatigue. Loss of tension then re-activates the SAC, thereby establishing a feed-forward loop (Fig. 7). It also indicates that apcin alone can only partially inhibit APC/C activity during mitosis. This could be advantageous in therapy, because basal APC/C function permits cell division of healthy somatic cells.

The increased apcin sensitivity could be translated to most cell lines with cohesion defects. It should be noted, however, that apcin did not inhibit growth of all cohesion-defective cells. Although numerous pharmacological and intracellular factors could influence the drug response, this observation may also indicate that not every condition that we characterize as ‘defective cohesion' in metaphase spreads leads to a substantial acceleration of the process ‘cohesion fatigue'. The precise nature of defective sister chromatid cohesion, such as those arising from reduced total levels of cohesive rings, their improper distribution along chromosomes or disturbed chromatin organization, may be relevant. Cohesion defects may result from replicative stress, such as those found in pRB-negative cells^59^, so they could occur even more frequently than currently anticipated based on common mutations of known cohesin factors^1^. Pharmacological inhibition of the APC/C has long been considered unfeasible in a clinical setting. However, here we identified defective sister chromatid cohesion, an emerging hall mark of many tumours, as a novel foothold for cancer therapy by APC/C inhibitors. Future work will need to be directed at finding biomarkers of cohesion defects, that might predict response to apcin, and testing the effects of apcin or other APC/C inhibitors in animal cancer models.

## Methods

### Cell lines and drug treatments

Human fibroblasts derived from a previously described WABS patient^8^ were immortalized with hTERT and SV40 large T antigen, stably transfected with DDX11 cDNA or an empty vector, and single colonies were analysed for DDX11 protein levels. The official names of the resulting cell lines are VU1149+SV40+DDX11 and VU1149+SV40+pcDNA, however for clarity they were renamed DDX11^+^ and DDX11^−^ in this manuscript. Wild-type fibroblasts LN9SV and RBS fibroblasts VU1199+SV40 (ESCO2^−^) and VU1199+SV40+V5-ESCO2 (ESCO2^+^) have been described before^40^. HNSCC cell line VU-SCC-9917 was established from an HPV-negative T2N2B tumour in the oral cavity of a 62-year old woman. HNSCC cell lines VU-SCC-120, VU-SCC-147, VU-SCC-78 and VU-SCC-40 were described previously^60^. Luminal breast cancer cell lines MCF7, CAMA-1, OCUB-M and Sum185PE were kindly provided by J. Martens, Erasmus MC Rotterdam, Netherlands. MCF7 is listed in the database of commonly misidentified cell lines, ICLAC. The authenticity was assessed by comparing the generated Short Tandem Repeat (STR) profile with the source STR profiles present in the American Type Culture Collection and the Deutsche Sammlung von Mikroorganismen und Zellkulturen^61^. Human fibroblasts, human Retinal Pigment Epithelial cells (RPE1) as well as the cancer cell lines used in this study were cultured in Dulbecco's Modified Eagle's Medium (DMEM, GIBCO) with 10% FBS (Hyclone) and 1% L-glutamine (Invitrogen). The Mps1 inhibitor reversine as well as the spindle poisons paclitaxel (taxol) and nocodazole were purchased from Sigma -Aldrich. The APC/C inhibitor apcin has been recently described^41^.

The APC/C inhibitor proTAME, which causes cohesion fatigue under certain conditions^22^, could not be used in the long term cell viability assays that we describe here, due to variations in the stability of this APC/C inhibitor under cell culture conditions.

### Genome-wide siRNA screens

The DDX11^−^ and DDX11^+^ cell lines were subjected to a high-throughput reverse transfection protocol in 384-well tissue culture plates (Cellstar, Greiner Bio-One) using established automated liquid handling procedures. 1.5 pmol siRNA SMARTpools from the siARRAY Whole Human Genome library (Catalogue items G-003500 (Sept05), G-003600 (Sept05), G-004600 (Sept05) and G-005000 (Oct05); Dharmacon, Thermo Fisher Scientific) were dispensed into the wells and plates were stored in −20 °C. The non-targeting siControl#2 and the siPlk1 SMARTpool were used as controls. Lipofectamin RNAiMAX transfection reagent (Life Technologies) in OptiMem (GIBCO) was added to the wells using a Multidrop Combi (Thermo Fisher Scientific). After two hours, per well 500 cells in 40 μl growth medium were seeded to a final volume of 60 μl. Plates were incubated for 96 h at 37 °C/5% CO_2_. Cell viability was determined by adding 6 μl CellTiter-Blue reagent (Promega). After 4 h of incubation at 37 °C, the reaction was stopped by adding 30 μl 3% SDS and fluorescence (560_Ex_/590_Em_) was measured using an Infinite F200 microplate reader (Tecan). The potency of the selected hits was validated in a deconvolution screen: DDX11^−^ and DDX11^+^ cell lines were screened with four distinct siRNAs for each gene using a similar approach as described above.

### Analysis of screen data

Data were read into R and configured using the package cellHTS2 (ref. 62). Log-transformed intensities were normalized using a linear regression on data for all screens, correcting for experiment-wide plate and screen effects. This helps making plate averages across all screens the same, although individual plate averages may differ, as well as making screen averages the same across the entire experiment. This normalization preserves differences between screens, as it will affect all wells belonging to the same plate in the precise same way and as such it preserves the effect between cell lines under study. We then used an empirical-Bayes linear regression model^63^ to find siRNAs that led to differential cell growth in DDX11^−^ compared with DDX11^+^ cell lines. FDR-corrected *P* values were selected if they were at most 0.10, so it is expected that at most 10% of those selected are false positives^64^. This regression model is particularly suitable to handling data from experiments as this one, where a small number of samples is available per group (only 2 samples in one group and 3 in the other), and a large number of siRNAs is tested simultaneously.

### siRNA transfection and viability assay

We used a standard siRNA concentration of 25 nM, except for the co-depletions in RPE1 cells, for which we used 2.5 nM per single siRNA. The RNAiMAX dilution factor was optimized for each cell line separately: 1200 × for RPE1, DDX11^−^, DDX11^+^, UM-SCC-14B and UM-SCC-14C; 800 × for ESCO2^−^, ESCO2^+^, OCUB-M, MCF7 and CAMA-1. Unless differently stated, we used siRNA#4 for APC3 and the pool of four siRNAs for ESCO2, WAPL and Separase. In CellTiter-Blue assays, we used either siPlk1 or siUBB (Ubiquitin B) as positive control for transfection efficiency. The sequences of siRNAs that were used in the deconvolution screen are provided in Supplementary Data 2. In addition, the following sequences were used: non-targeting siRNA UAAGGCUAUGAAGAGAUAC; siPLK1 pool CAACCAAAGUCGAAUAUGA, CAAGAAGAAUGAAUACAGU, GAAGAUGUCCAUGGAAAUA, CAACACGCCUCAUCCUCUA; siUBB pool CCCAGUGACACCAUCGAAA, GACCAUCACUCUGGAGGUG, GUAUGCAGAUCUUCGUGAA, GCCGUACUCUUUCUGACUA; siAPC2 pool #5–8 GAGAUGAUCCAGCGUCUGU, GACAUCAUCACCCUCUAUA, GAUCGUAUCUACAACAUGC, GAGAAGAAGUCCACACUAU; siWAPL pool CAAACAGUGAAUCGAGUAA, CCAAUCAAGGGAUCUGUUA, GAAGGAGACUUUUCAAUAA, GCAAACACAUGGAGGAUUG; siSeparase pool CCGAGGAUCACUUGAAAUA, GGAGAAGGCUCACAGUUAC, GAUCGUUUCCUAUACAGUA, GGAACGAAUUCUCUUUGUC.

The viability assays in follow-up experiments were carried out in 96-wells plates. Cells were counted and seeded in at least triplicates in a total volume of 100 μl medium. Optimized cell densities were: DDX11^−^ 3,000/well, DDX11^+^ 3,000/well, ESCO2^−^ 4,000/well, ESCO2^+^ 4,000/well, SCC-99-17 3,000/well, VU120 3,000/well, VU147 8,000/well, MCF7 4,000/well, VU78 2,500/well, CAMA-1 4,000/well, OCUB-M 8,000/well, VU0040 8,000/well, Sum185 8,000/well, UM-SCC-14B 3,000/well, UM-SCC-14C 3,000/well. Cells were incubated with 10 μl CellTiter-Blue reagent (Promega) for 2–4 h and fluorescence (560_Ex_/590_Em_) was measured in a microplate reader (TriStar LB 941, Berthold Technologies).

### Immunoblotting

Proteins were isolated in lysis buffer (50 mM Tris-HCl pH 7.4, 150 mM NaCl, 1% Triton X-100) with protease- and phosphatase inhibitors, separated by 3–8% or 8–16% SDS-PAGE (NU-PAGE), blotted onto polyvinylidene fluoride transfer membranes, incubated with the appropriate primary and secondary antibodies, and bands were visualized by chemoluminescence (Amersham). Antibodies used for detection are mouse anti-DDX11 (Abnova #H00001663-B01P, dilution 1:1,000), mouse anti-vinculin (H-10, Santa Cruz #sc-25336, dilution 1:1,000), rabbit anti-APC2 (kind gift from J. Pines, dilution 1:5,00), mouse anti-α-tubulin (B-5-1-2, Santa Cruz #sc-23948, dilution 1:5,000), mouse anti-APC3 (BD Transduction laboratories, #610454, dilution 1:1,000), goat anti-APC4 (C-18, Santa Cruz #SC-21414, dilution 1:500), rabbit anti-p31^comet^ (Abcam #ab150363, dilution 1:1,000), guinea pig anti-ESCO2 (ref. 40) (dilution 1:1,000), mouse anti-Rad21 (Oncogene #NA80, dilution 1:1,000) and rabbit anti-WAPL (Bethyl #A300-268 A, dilution 1:1,000). Uncropped images of western blots are provided in Supplementary Fig. 9.

### Flow cytometry

Cells were harvested, washed in PBS and fixed in ice-cold 70% EtOH. For mitosis detection, cells were incubated with rabbit anti-pS10-Histone H3 (Millipore) for 1 h and with Alexa Fluor 488 goat-anti-rabbit (Invitrogen) for 30 min. Cells were washed and resuspended in PBS with 1:10 PI/RNase staining buffer (BD Biosciences) and analysed by flow cytometry on a BD FACSCalibur (BD Biosciences). Cell cycle analysis was conducted with BD CellQuest software (BD Biosciences).

### Time-lapse microscopy

Cells were seeded in a 35 mm glass-bottom dish (Willcowells). Acquisition of DIC images started 48 h post-transfection on a microscope (Axio Observer Z1; Carl Zeiss) in a heated culture chamber (5% CO2 at 37 °C). The microscope was equipped with an LD 0.55 condenser and × 40 NA 1.40 Plan Apochromat oil DIC objective. Images were taken using AxioVision Rel. 4.8.1 software (Carl Zeiss) with a charge-coupled device camera (ORCA R2 Black and White CCD [Hamamatsu Photonics] or Roper HQ [Roper Scientific]) at 100-ms exposure times. Images were analysed using MetaMorph software (Universal Imaging).

### Cohesion defect analysis

For cohesion defect analysis, cells were incubated with 200 ng ml^−1^ Demecolcin (Sigma-Aldrich) in medium for 20 min, harvested, resuspended in 75 mM KCl for 20 min and fixed in methanol/acetic acid (3:1). Cells were dropped onto glass slides, stained with 5% Giemsa (Merck) and cohesion defects were microscopically analysed. Per condition, 25 metaphases per slide were counted on two coded slides as technical replicate. For coding, we covered the text, randomly distributed the slides on the bench and numbered the slides in random order.

## Additional information

**How to cite this article:** De Lange, J. *et al*. Defective sister chromatid cohesion is synthetically lethal with impaired APC/C function. *Nat. Commun.* 6:8399 doi: 10.1038/ncomms9399 (2015).

## Supplementary Material

Supplementary Information Supplementary Figures 1-9

Supplementary Data 1 Genome-wide siRNA screens in DDX11- and DDX11+ cells. Regression-normalized data, p-values and False Discovery Rates (FDR) of all screens described in Fig 1b, produced in R using the cellHTS2 software package. In addition, we calculated the mean viability and standard deviations for each siRNA relative to 8 nontargeting control siRNAs present on the same plate.

Supplementary Data 2 Deconvolution of 98 siRNA's in DDX11- and DDX11+ cells. Two independent deconvolution experiments were performed in both cell lines using 98 hits of the initial screen. For every single siRNA, the gene accession number, target sequence and % viability relative to a non-targeting control siRNA for both screens are shown. Mean viabilities were used to calculate the ratio (DDX11+ / DDX11-) and toxicity (% cell death in DDX11+ cells) for each siRNA. The genes for which at least two out of four siRNAs show ratio>2 and toxicity <50% were considered 'confirmed'.

Supplementary Movie 1 Example of a normal anaphase (H2B-GFP).

Supplementary Movie 2 Example of a normal anaphase (DIC).

Supplementary Movie 3 Example of chromosome scattering followed by cell division (H2B-GFP).

Supplementary Movie 4 Example of chromosome scattering followed by cell division (DIC).

Supplementary Movie 5 Example of chromosome scattering followed by mitotic death (H2B-GFP).

Supplementary Movie 6 Example of chromosome scattering followed by mitotic death (DIC).
