# Supplementary material for: Defective sister chromatid cohesion is synthetically lethal with impaired APC/C function
Source: Nat Commun. 2015 Oct 1;6:8399. doi: 10.1038/ncomms9399 (PMC4600715; doi:10.1038/ncomms9399)

Supplementary Figure 1: Statistical analysis of screen data.

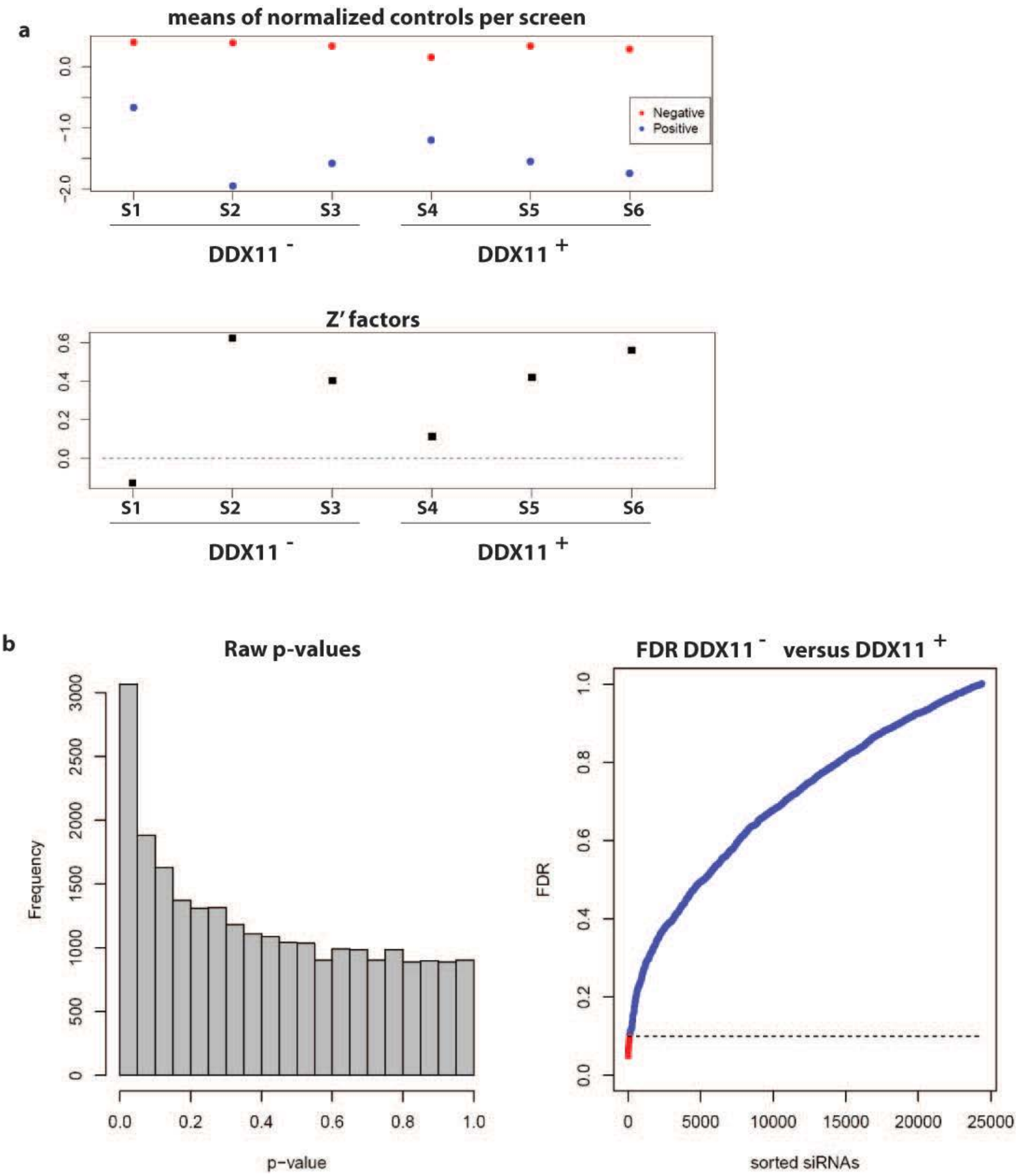

(**a**) Means of controls (top) and Z' factors (bottom) calculated for the log2-normalized data of all six screens. Per screen and control type (positive, negative) a total of 544 values are used. The negative control means are stable across screens, but the positive control means vary more. Screen 1 produced a relatively high positive control mean and, as a result, a Z' factor below 0. This was caused by poor cell growth in this particular case and therefore, this screen was excluded from further analysis. (**b**) Left: P-values computed per siRNA, for the difference between $DDX11^-$ and $DDX11^+$ cell lines. The enrichment of p-values near zero suggests a subset of siRNAs produces different results between the two cell lines. Right: FDRs corresponding to the p-values, where the number selected can be easily read for each FDR-control level. At the chosen control level of 0.10, 113 siRNAs are selected, of which 11 may be false positives.

Supplementary Figure 2: **Increased sensitivity of DDX11- cells to APC2 knockdown.**

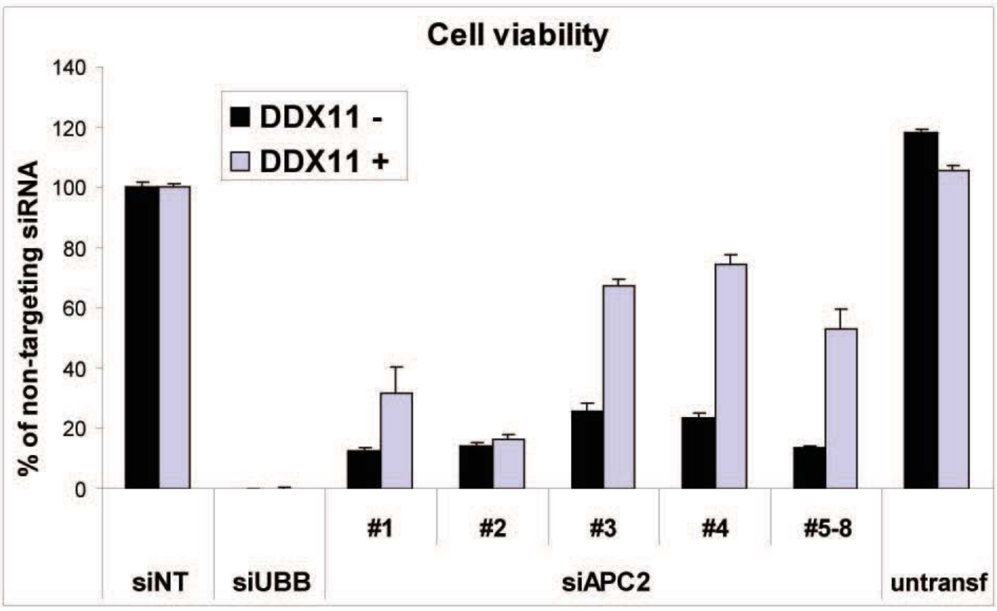

Cells were transfected with a pool of four APC2 siRNAs (#5-8, which do not overlap with the sequences of siAPC2 #1-4 used in Fig. 1c). Viability was measured at day 4 with a CellTiter-Blue assay.

Supplementary Figure 3: **ESCO2 mutation sensitizes to siRNA-mediated APC/C inhibition.**

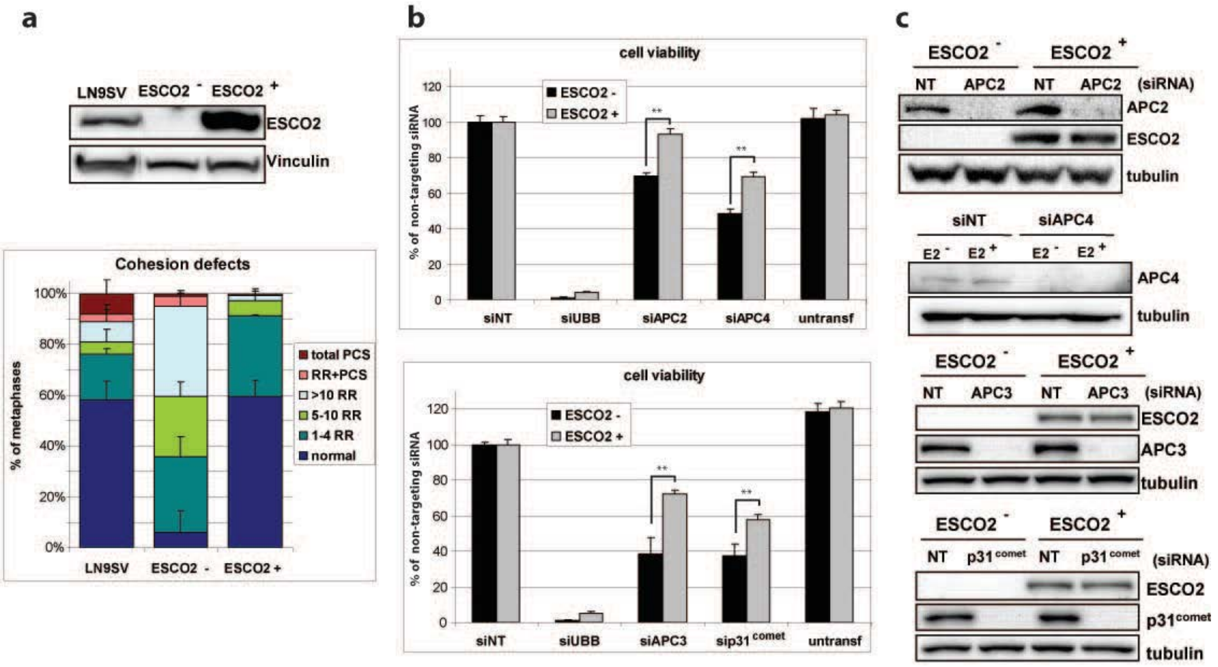

(**a**) Western blot and cohesion defect analysis of ESCO2⁻ and ESCO2⁺ cells next to SV40-immortalized wild-type LN9SV cells. Error bars denote standard deviations of two independent experiments. Two irrelevant lanes between lane 1 and 2 were spliced out. (**b,c**) Cells were transfected with the indicated siRNAs. Protein levels were analyzed after three days by western blot and cell viability was measured after five days using a CellTiter-Blue assay. Error bars denote standard deviations of at least three technical replicates.

Supplementary Figure 4: **DDX11 mutation sensitizes to pharmacological APC/C inhibition.**

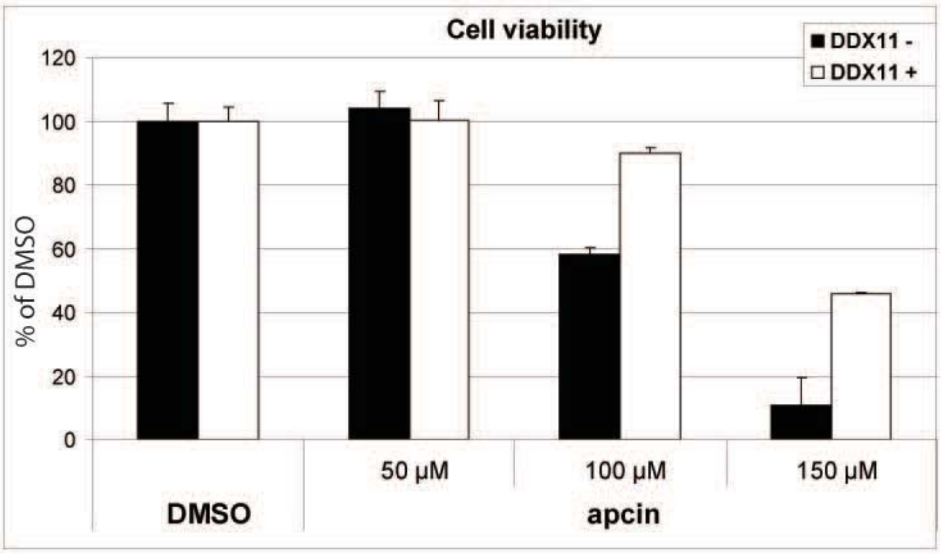

DDX11^{-} and DDX11^{+} cells were treated with different concentrations of apcin for three days and cell viability was measured using a CellTiter-Blue assay. Error bars denote standard deviations of eight technical replicates.

Supplementary Figure 5: Increased 4N DNA content upon knockdown of APC/C components.

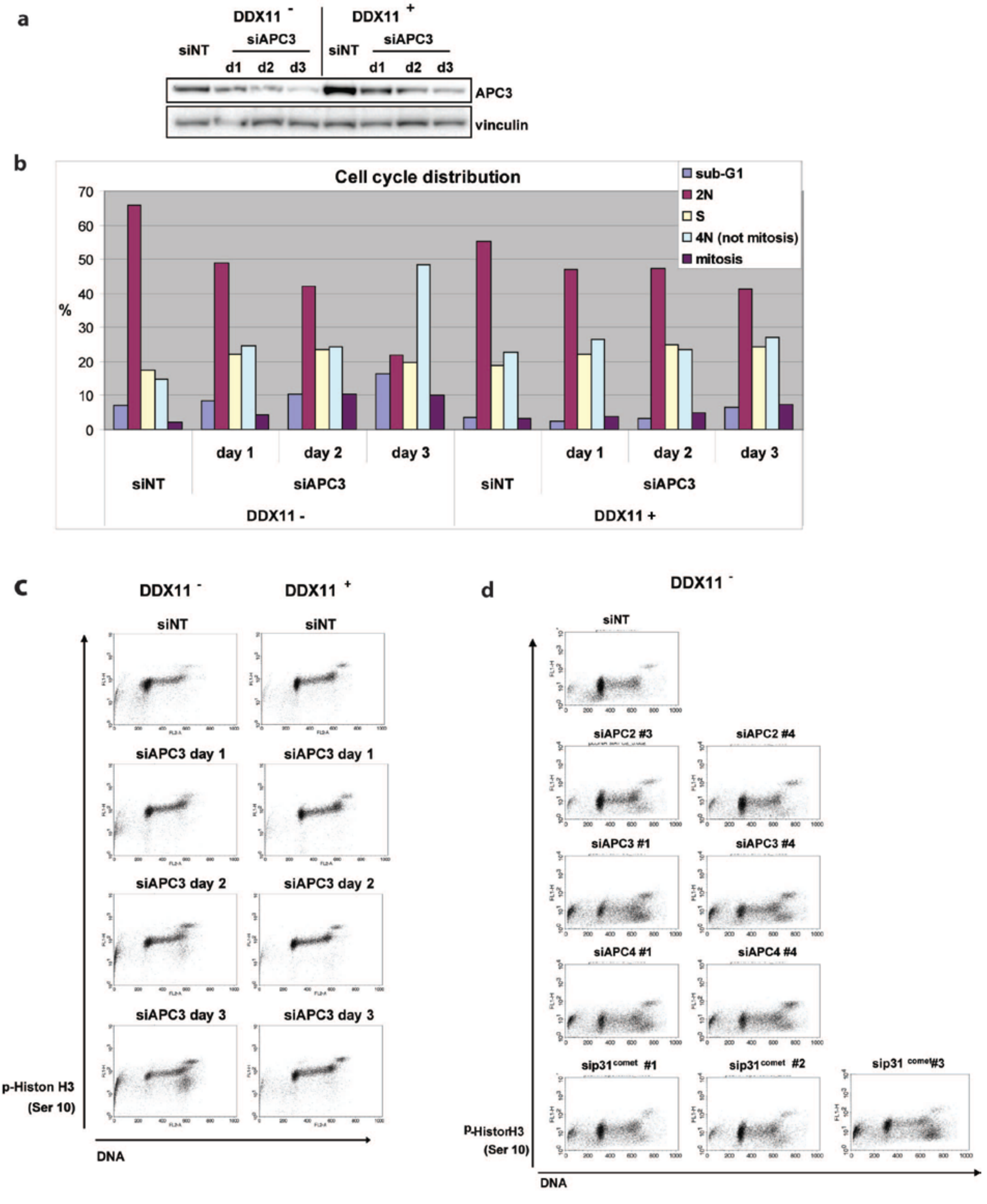

(**a-c**) Cells were harvested at different time-points after siAPC3 transfection and analyzed by western blot and flow cytometry using co-staining of propidium iodide and phospho-Histone H3. DDX11^{-} cells were transfected with indicated siRNAs, harvested after three days and analyzed by flow cytometry.

Supplementary Figure 6: Synthetic lethality of Rad21 and APC3, as well as of DDX11 and APC3.

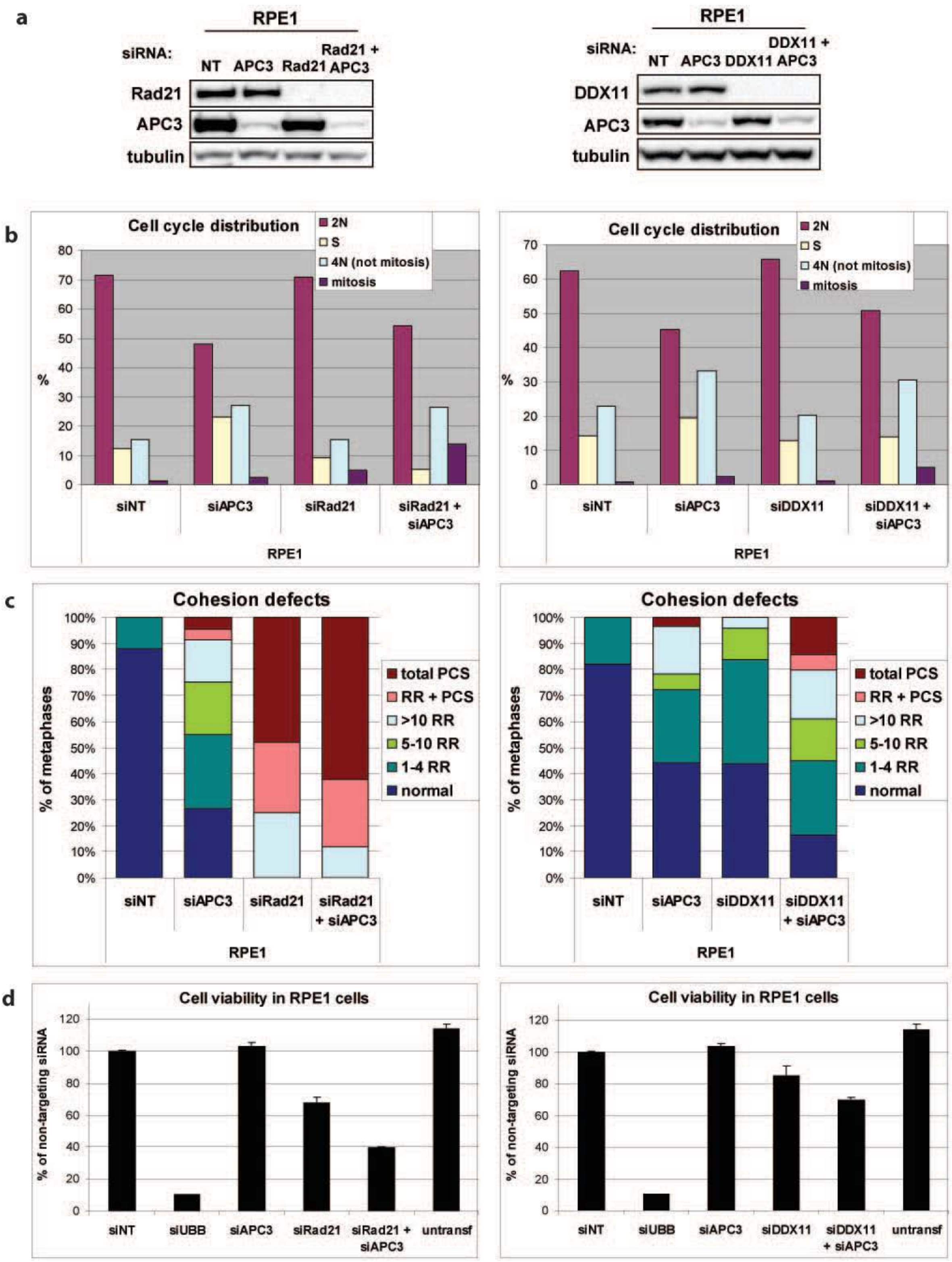

RPE1 cells were transfected with the indicated siRNAs. Western blot (a) flow cytometry (b) and cohesion defect analysis (c) were performed two days after transfection. Viability was measured at day four with a CellTiter-Blue assay (d). Error bars denote standard deviations of three technical replicates.

Supplementary Figure 7: Titrating siRNA concentration confirms differential sensitivity of DDX11$^-$ and DDX11$^+$ cells to APC3 inhibition.

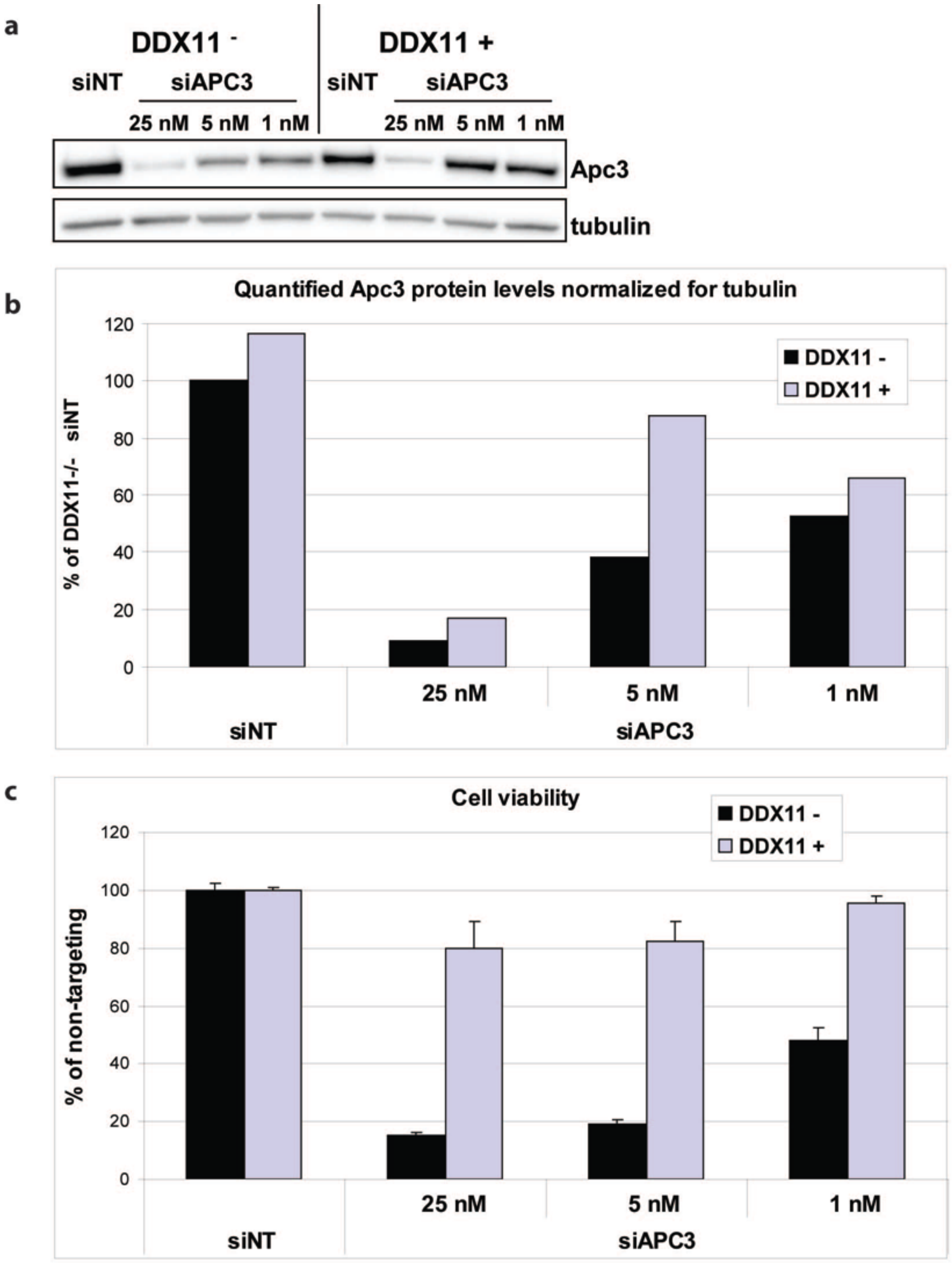

Cells were transfected with different concentrations of APC3 siRNA and protein levels were analyzed by western blot (a). Bands were quantified and normalized to tubulin (b). Cell viability was measured with a CellTiter-Blue assay (c). Note that 1 nM siAPC3 in DDX11$^-$ cells causes a much weaker knockdown than 25 nM in DDX11$^+$ cells, while the effect on viability is still stronger in DDX11$^-$ cells.

Supplementary Figure 8: Responses to APC/C inhibition are dependent on th spindle assembly checkpoint.

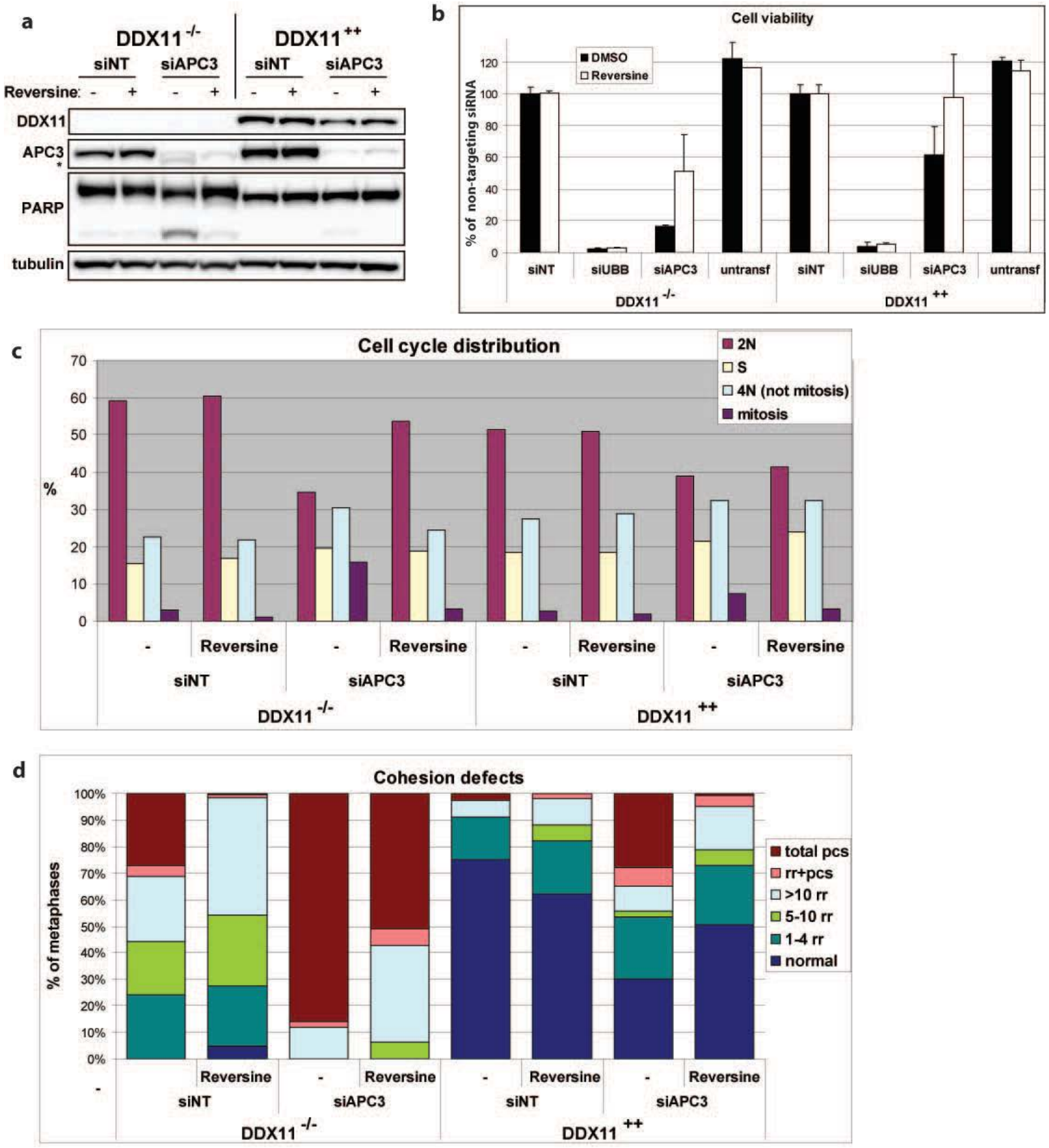

Cells were transfected with the indicated siRNAs and after 1 day 100 nM reversine or DMSO was added. Western blot, flow cytometry and cohesion defect analysis were performed two days after transfection and cell viability was measured four days after transfection. Error bars denote standard deviations of at least three technical replicates. The asterisk indicates detection of residual cleaved PARP signal in the APC3 blot. Note that the data for DDX11- cells are also shown in Fig. 5a-d.

Supplementary Figure 9: Uncropped western blots of main figure experiments

Figure 1a

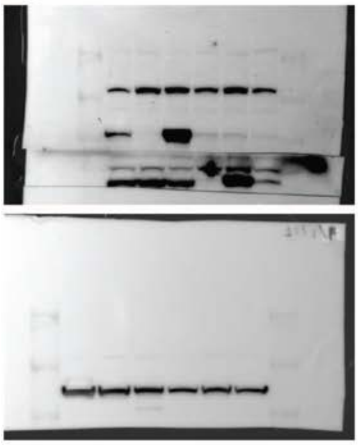

Figure 1d

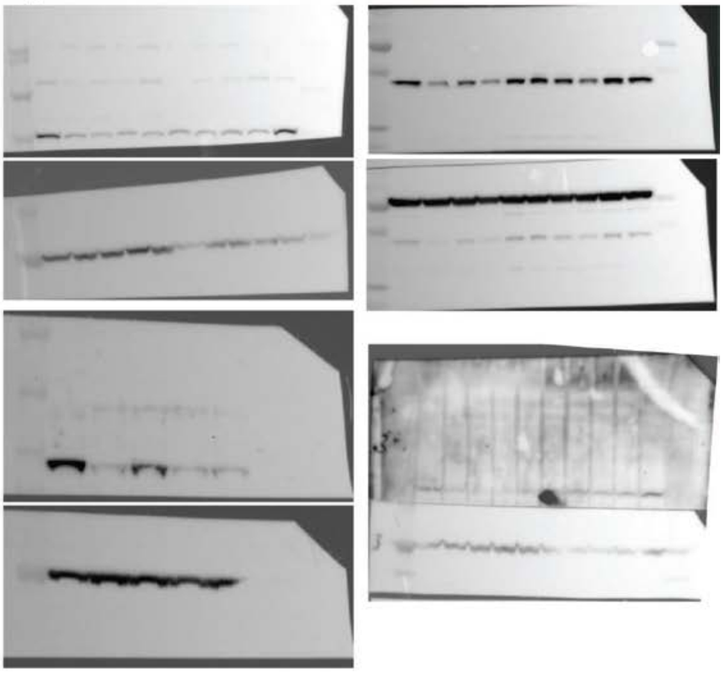

Figure 2c

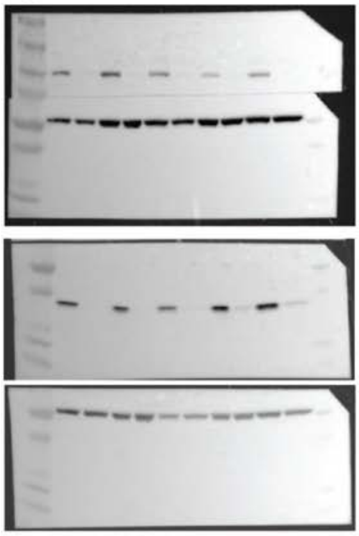

Figure 4a

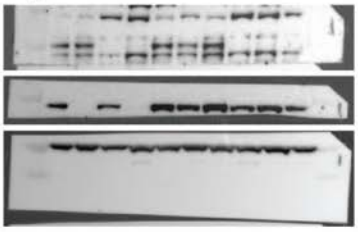

Figure 4e

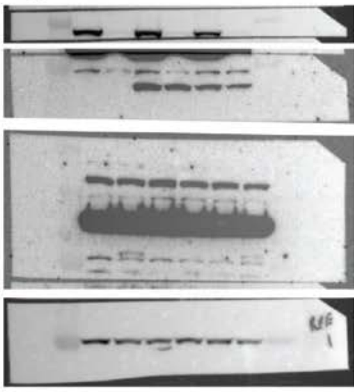

Figure 5a

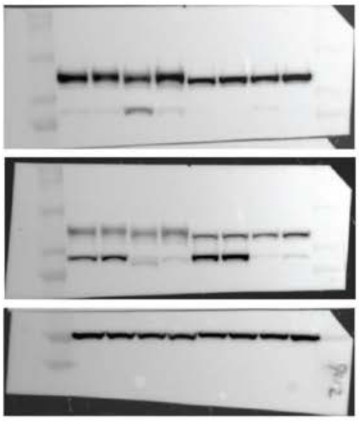

Figure 5f

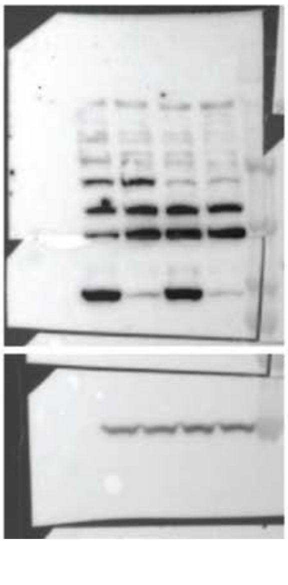

Supplement: Supplementary Information — Supplementary Figures 1-9 [file ncomms9399-s1.pdf]
